# Supplementary material for: Chloramphenicol-mobilized Bacillus subtilis transiently expresses resistance to multiple antibiotics, including the glycopeptides phleomycin and bleomycin
Source: J Bacteriol. 2026 Sep 18;208(10):e00452-26. doi: 10.1128/jb.00452-26 (PMC13689557; doi:10.1128/jb.00452-26)
Supplement: Supplementary file 1 [file jb.00452-26-s0001.pdf]

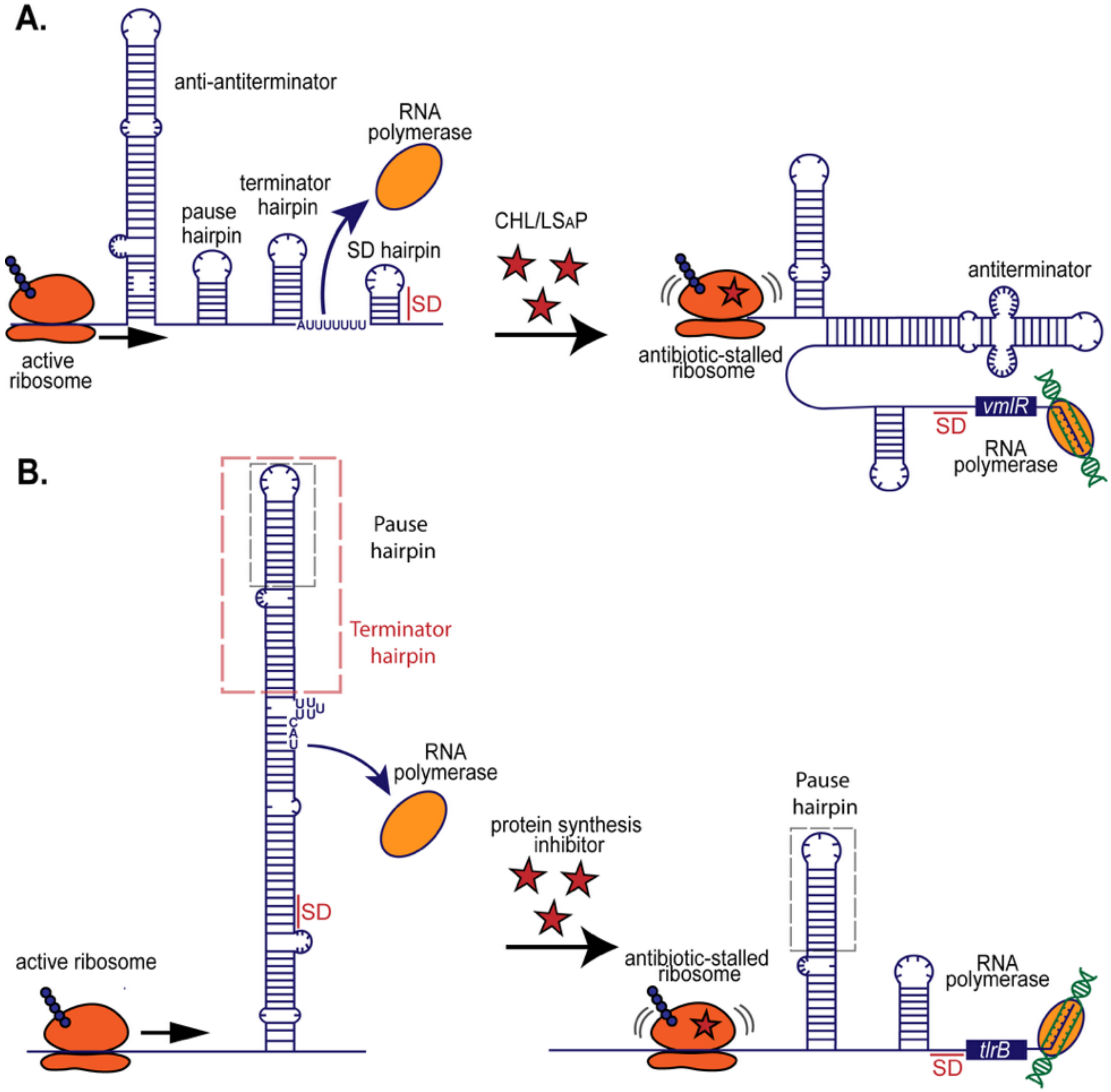

Figure S1. Models for VmlR and TlrB regulation Overview of the leader peptide transcription attenuation regulatory mechanisms for *vmlR* (**A**) and *tlrB* (**B**). **A.** In the absence of drug, the leader peptide nascent transcript forms 4 stem loops: an anti-antiterminator hairpin, a NusG-dependent pause hairpin, a terminator hairpin, and, if there is leaky transcription past the terminator hairpin, a Shine-Dalgarno-sequestering (SD) hairpin that prevents ribosome binding. Expression of *vmlR* is induced in the presence of CHL or L_SAPs. These protein synthesis inhibitors stall the ribosome causing only partial formation of the first stem loop and formation of antiterminator structure. Therefore, transcription proceeds to the VmlR ORF (15). **B.** The regulation of *trlB* is more complex than *bmrCD* and *vmlR*. In the absence of antibiotic, NusG-dependent pausing allows for initiation of *tlrB* uORF translation. The ribosome interferes with the formation of a terminator hairpin when reaching the uORF stop codon, but then releases, and

termination occurs. Transcripts evading termination are suppressed due to the stem loop sequestering the *tlrB* SD sequence. In the presence of tylosin or other translation inhibitors, however, the ribosome stalls on the uORF where the terminator hairpin would begin to form. Transcription termination is therefore suppressed. Additionally, the SD sequence is no longer trapped within a stem loop structure, and the ribosome can bind the transcript. Therefore, *tlrB* is transcribed and subsequently translated (12).

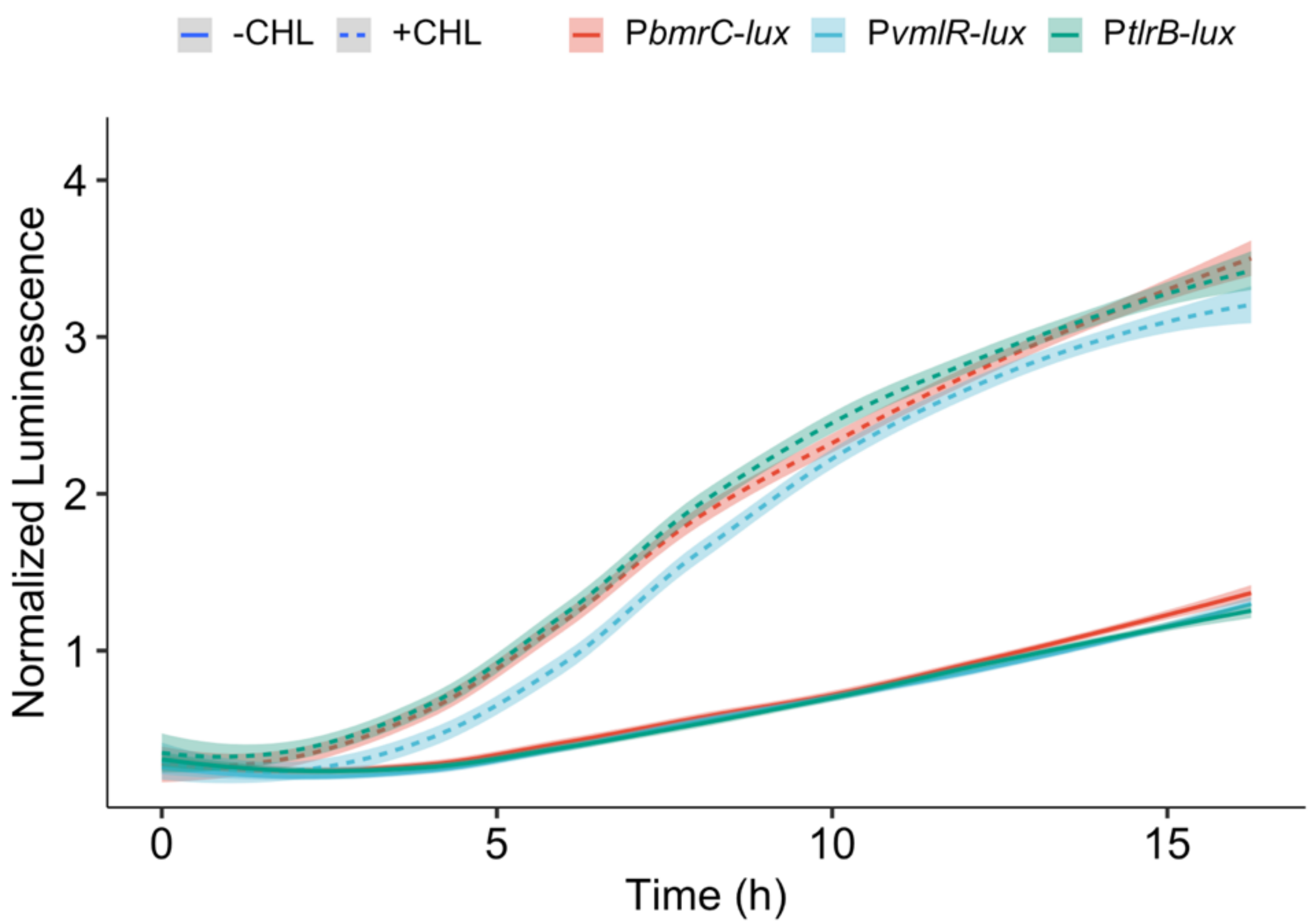

Figure S2. Transcriptional activity of 5'uORF-luciferase fusions P*bmrC-lux,* P*vmlR-lux,* and P*tlrB-lux* in relative light units (RLU) exposed to 1 μM CHL (dashed) compared to non-exposed (solid) grown over 16 hours. The curves were normalized to a luciferase construct lacking a promoter.

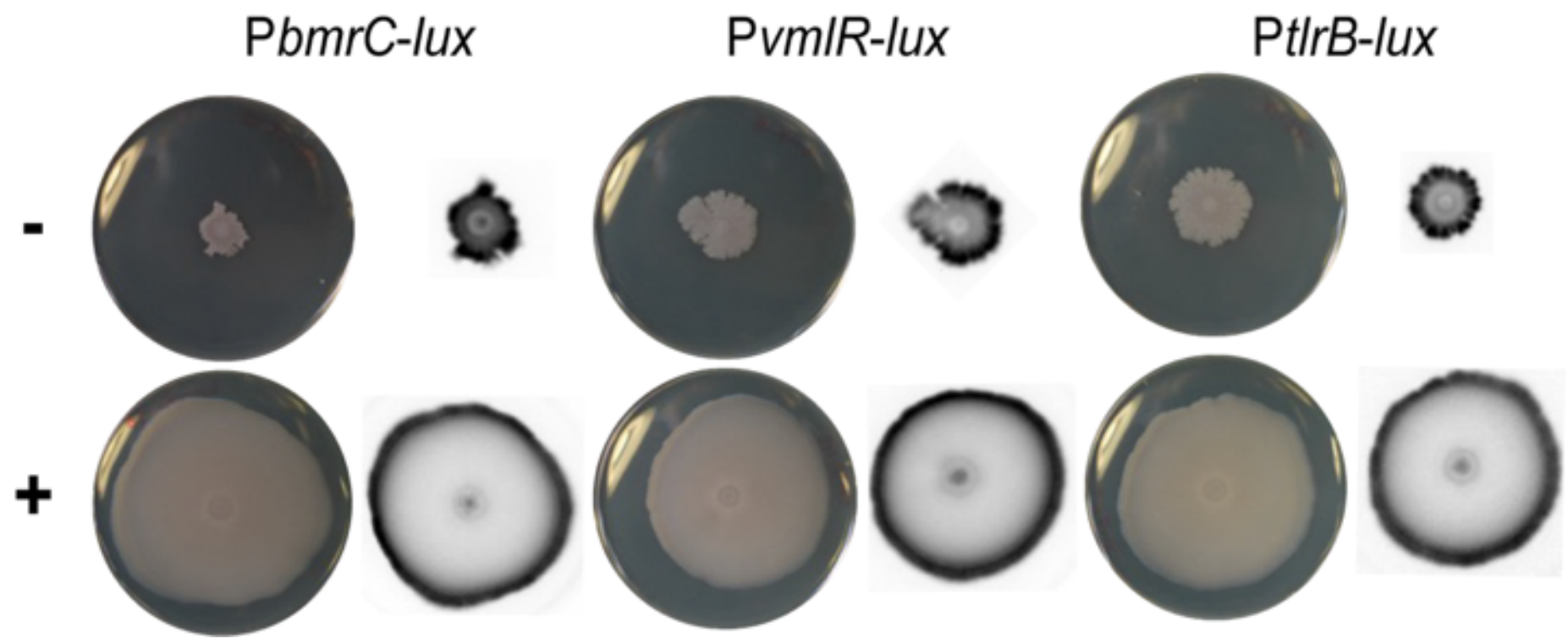

Figure S3. Transcriptional activity of resistance genes at 48 hours of growth (**Note:** The chemiluminescence images are inverted. The luminescence signal appears gray to black with increasing intensity) P*bmrC-lux,* P*vmlR-lux,* and *PtlrB-lux* spotted on agar plates with 1 μM CHL (+) or without CHL (-). Pictures were taken with phase contrast (left) and chemiluminescence (right) at 48 hours of growth.

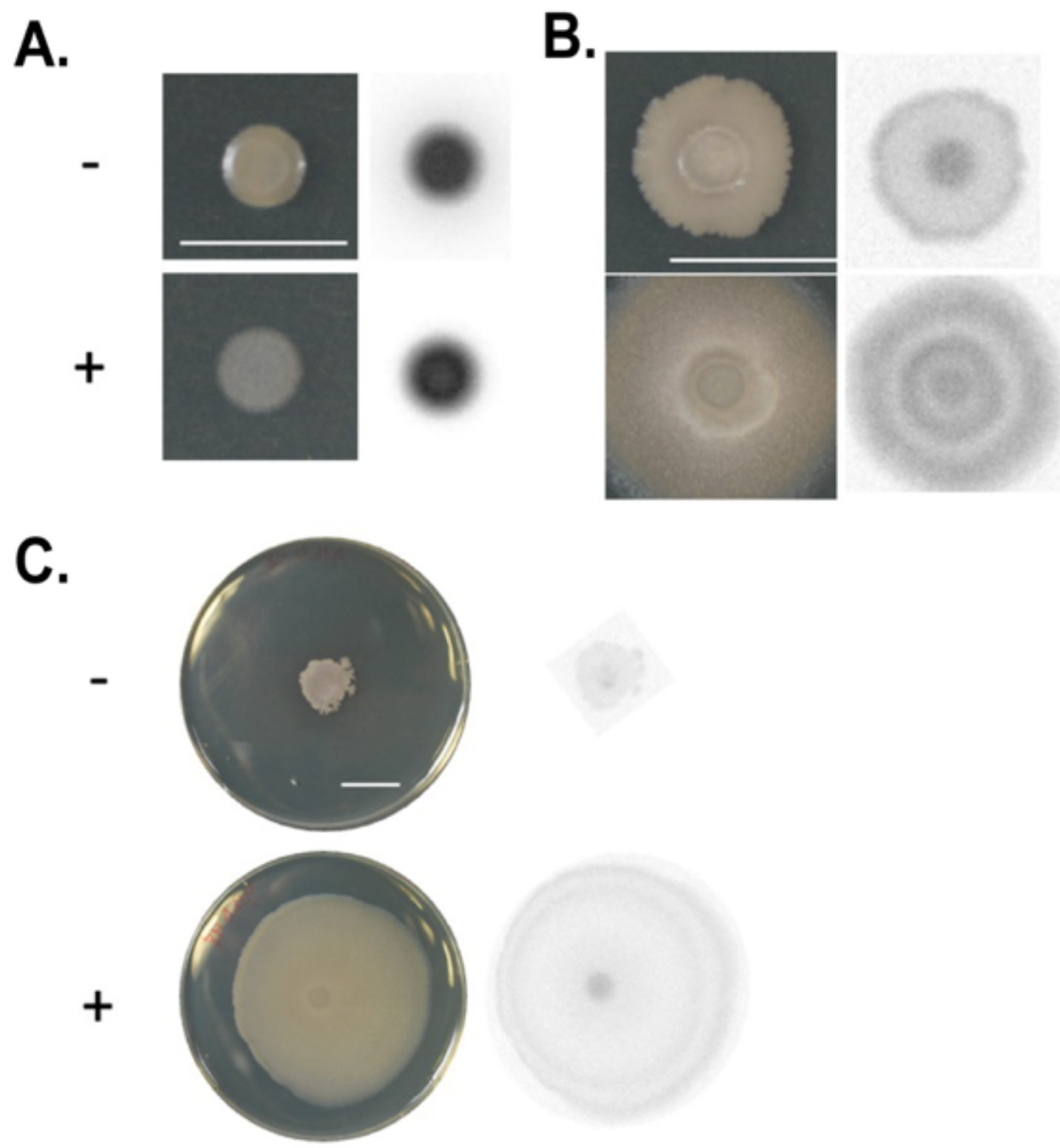

Figure S4. Transcriptional activity of *ytbDE* over 24 hours of growth (**Note:** The chemiluminescence images are inverted. The luminescence signal appears gray to black with increasing intensity) P*ytbDE-lux* was spotted on agar plates with 1 μM CHL (+) or without CHL (-). Pictures were taken with phase contrast (left) and chemiluminescence (right) at **A.** 6 hours, **B.** 24 hours, and **C.** 48 hours of growth.

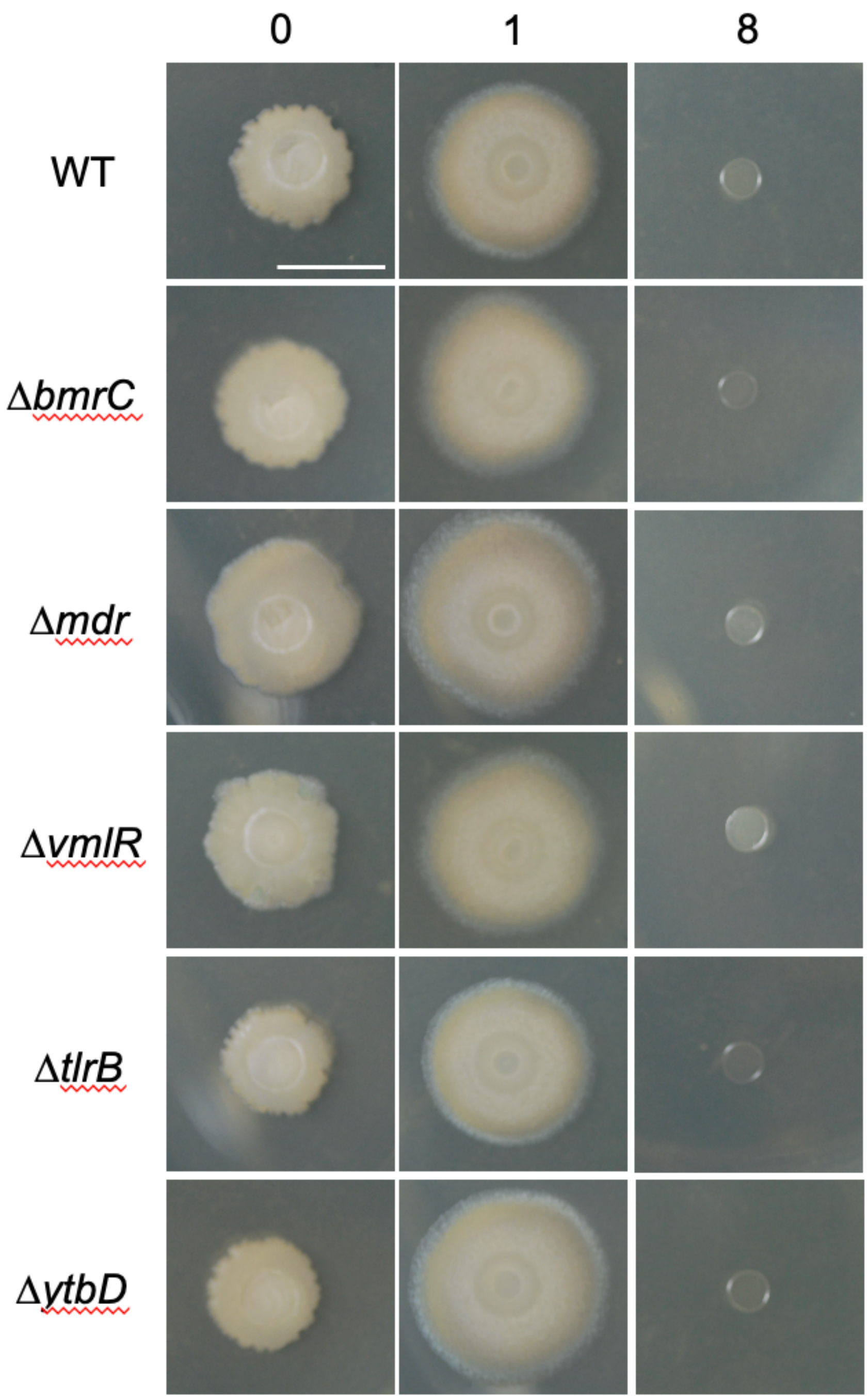

Figure S5. Single gene deletions of each resistance gene do not provide resistance or change colony morphology in response to CHL exposure. Wild type (WT) and each deletion strain were plated on the GYM7 plate with no CHL, subinhibitory CHL (1 μM) and inhibitory CHL (8 μM). Pictures were taken at 24 h.

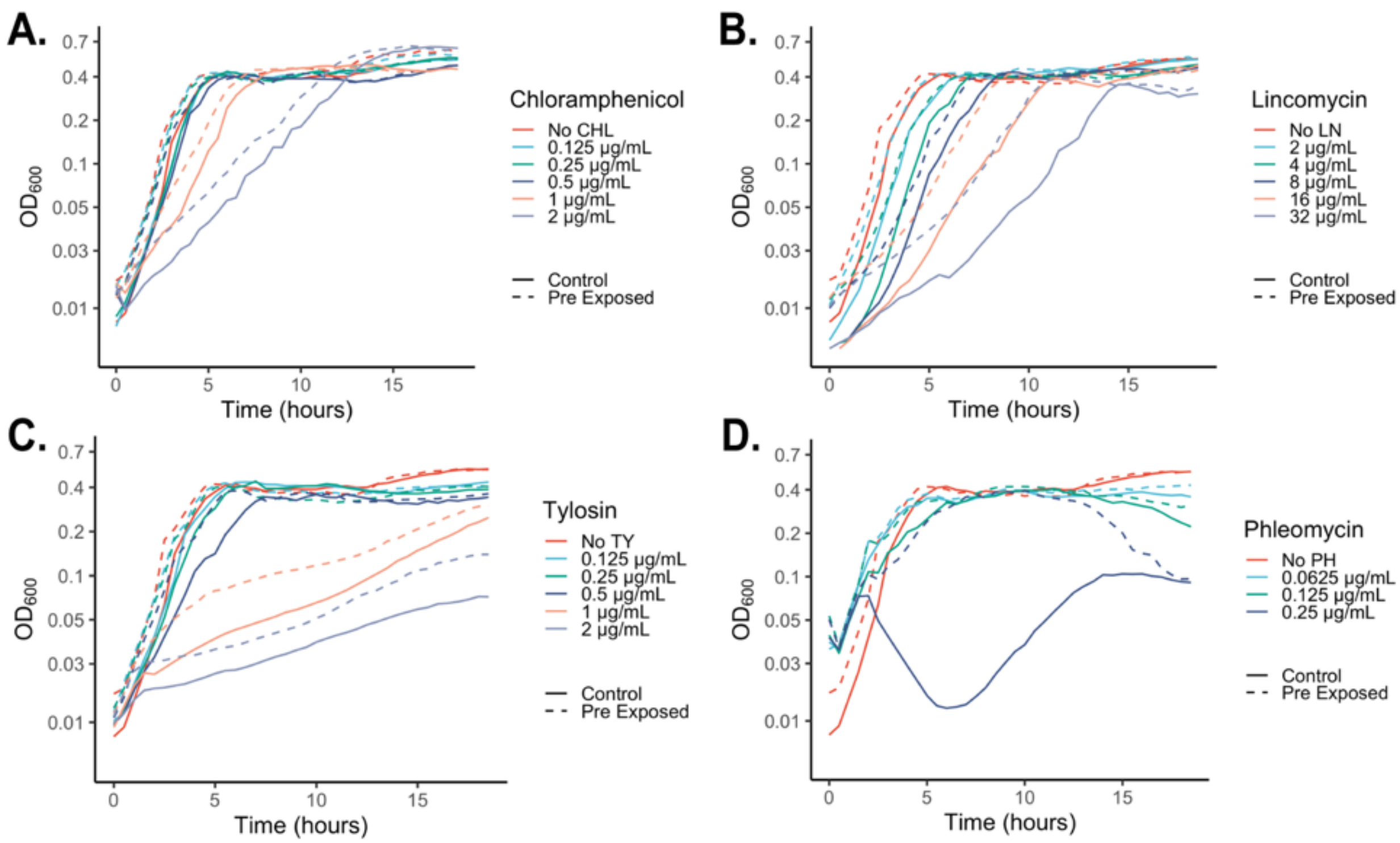

Figure S6. Growth comparison of cultures pre-exposed to CHL (dashed) to cultures grown in isolation (solid) at a range of (**A**) Chloramphenicol, (**B**) Lincomycin, (**C**) Tylosin, and (**D**) Phleomycin concentrations over 18 hours of growth. OD_{600} values were taken every 30 minutes. Each line is an average of 4 replicates.

BmrC

MFSVLKKLGW FFKAYWLRYT IAIVLLLAVN VIEMFPPKLL GNAIDDMKAG AFTAEGLLFY IGIFFVLTAA VYIMSYFWMH QLFGGANLME KILRTKLMGH

LLTMSPPFYE KNRTGDLMAR GTNDLQAVSL TTGFGILTLV DSTMFMMTIF LTMGFLISWK LTFAAIIPLP VMAIAISLYG SKIHERFTEA QNAFGALNDR

VLESVSGVRV IRAYVQETND VRRFNEMTAD VYQKNMKVAF IDSLFEPTVK LLVGASYLIG LGYGAFLVFR NELTLGELVS FNVYLGMMIW PMFAIGELIN

Walker A

VMQRGNASLD RVNETLSYET DVTDPKQPAD LKEPGDIVFS HVSFTYPSST SDNLQDISFT VRKGQTVGIA GKTGSGKTTI IKQLLRQYPP GEGSITFSGV

*

Walker B

PIQQIPLDRL RGWIGYVPQD HLLFSRTVKE NILYGKQDAT DKEVQQAIAE AHFEKDLHML PSGLETMVGE KGVALSGGQK QRISIARALM ANPEILILDD

SLSAVDAKTE AAIIKNIREN RKGKTTFILT HRLSAVEHAD LILVMDGGVI AERGTHQELL ANNGWYREQY ERQQLFTAEE GGAGA

BmrD

MKIGKTLWRY ALLYRKLLIT AVLLLTVAVG AELTGPFIGK KMIDDHILGI EKTWYEAAEK DKNAVQFHGV SYVREDRLQE PVSKAKEAHI YQVGMAFYFV

DQAVSFDGNR TVSDGKLTIT NGDKSRAYAA EKLTKQELFQ FYQPEIKGMV LLICLYGGLL VFSVFFQYGQ HYLLQMSANR IIQKMRQDVF SHIQKMPIRY

FDNLPAGKVV ARITNDTEAI RDLYVTVLST FVTSGIYMFG IFTALFLLDV KLAFVCLAIV PIIWLWSVIY RRYASYYNQK IRSINSDINA KMNESIQGMT

IIQAFRHQKE TMREFEELNE SHFYFQNRML NLNSLMSHNL VNVIRNLAFV CLIWHFGGAS LNAAGIVSIG VLYAFVDYLN RLFQPITGIV NQFSKLELAR

Walker A

VSAGRVFELL EEKNTEEAGE PAKERALGRV EFRDVSFAYQ EGEEVLKHIS FTAQKGETVA LVGHTGSGKS SILNLLFRFY DAQKGDVLID GKSIYNMSRQ

*

Walker B

ELRSHMGIVL QDPYLFSGTI GSNVSLDDER MTEEEIKNAL RQVGAEPLLK KLPKGINEPV IEKGSTLSSG ERQLISFARA LAFDPAILIL DEATAHIDTE

TEAVIQKALD VVKQGRTTFV IAHRLSTIRN ADQILVLDKG EIVERGNHEE LMALEGQYYQ MYELQKGQKH SIA

Figure S7. Walker A and B motifs of BmrCD. Walker A and Walker B motifs are highly conserved nucleotide-binding sequences. BmrC and BmrD protein sequences with the Walker motifs highlighted in yellow. The sites mutated in this study (BmrC K377A; BmrD K469A) are indicated below the residue with an asterisk. These mutations prevent ATP binding, therefore preventing BmrCD from functioning as a multidrug efflux pump.

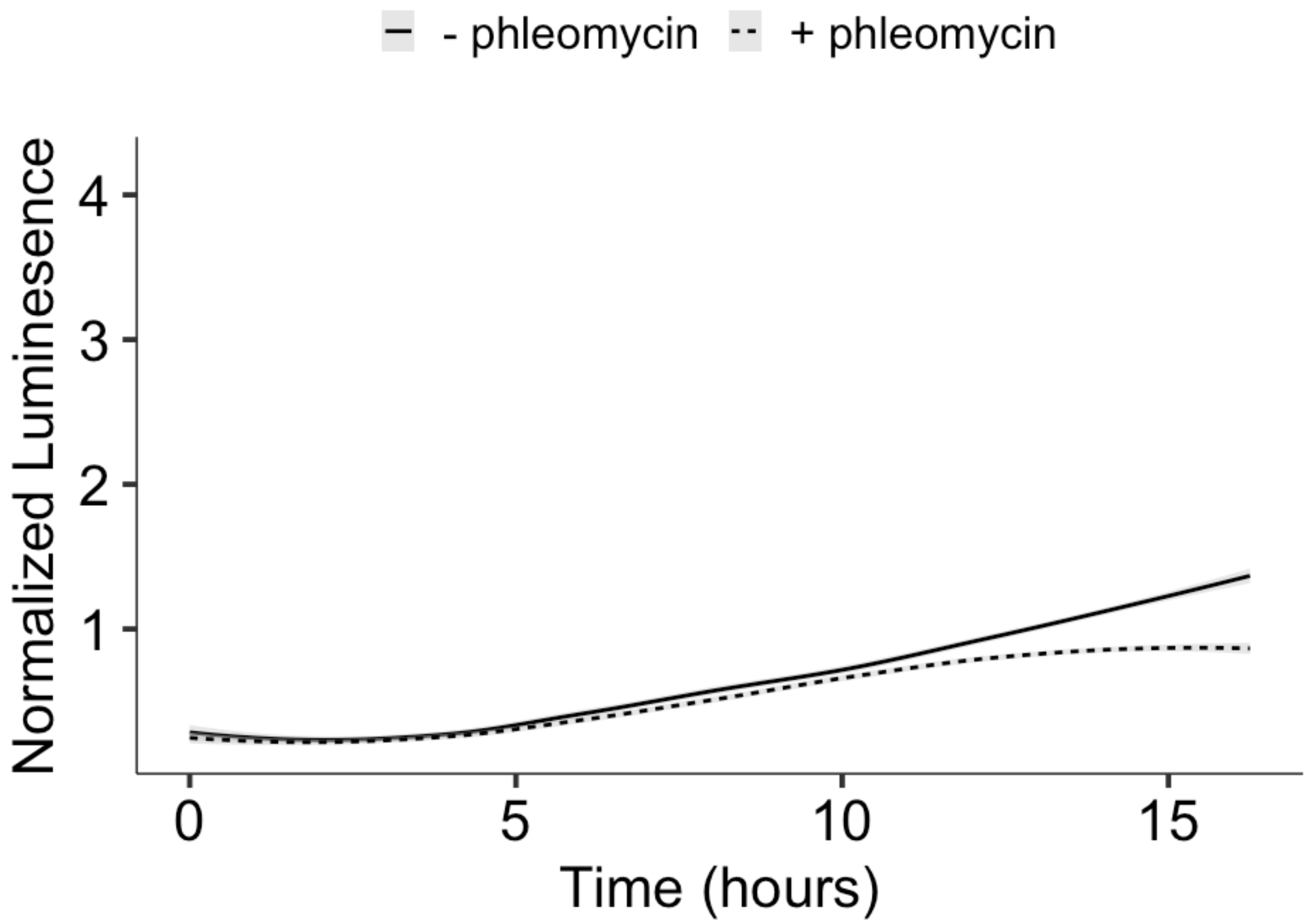

Figure S8. BmrCD expression is not triggered by phleomycin exposure. We monitored luminescence over 16 hours of P*bmrC-lux* grown with (dashed) and without (solid) 0.125 μg/mL phleomycin. Curves are normalized to a control luciferase construct lacking a promoter. Each line is average of 4 replicates.

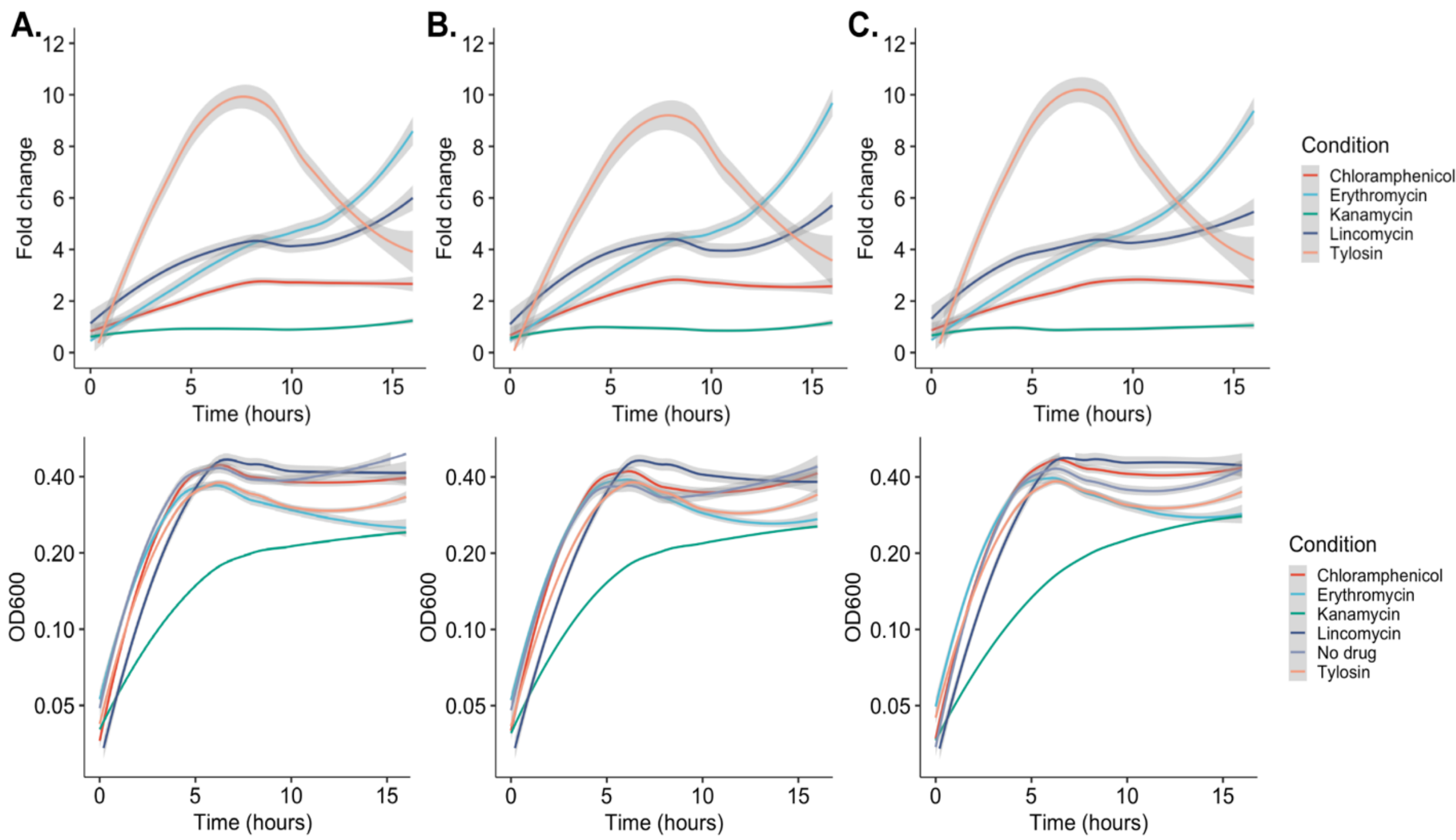

Figure S9. The expression of *bmrCD, vmlR,* and *tlrB* is induced by erythromycin, lincomycin, and tylosin exposure, but not kanamycin exposure. The fold change in relative light units (RLU) of (**A**) P*bmrC-lux,* (**B**) P*vmlR-lux,* and (**C**) *PtlrB-lux* exposed to 1 μM CHL, 0.05 μg/mL erythromycin, 1 μg/mL kanamycin, 2.5 μg/mL lincomycin, and 0.5 μg/mL tylosin compared to non-exposed reporter strains grown over 16 hours. The curves were normalized to a luciferase construct lacking a promoter. Below each expression plot are growth curves for each respective reporter strain exposed to each antibiotic.

Table S1. Antibiotics tested for resistance via *bmrCD and ytbDE*.

Antibiotic	Class
Apramycin	Aminoglycoside
Kanamycin	Aminoglycoside
Hygromycin B*	Aminoglycoside
Erythromycin	Macrolide
Tylosin*	Macrolide
Spectinomycin	Aminocyclitol
Ampicillin*	Penicillin
Carbenicillin*	Penicillin
Lincomycin	Lincosamide
Tetracycline	Tetracycline
Chloramphenicol	Amphenicol
Rifamycin**	Ansamycin
Phleomycin**	Glycopeptide

**Only tested for ytbDE*

***Only tested for bmrCD*

Table S2. Bacterial strains used in this study

Strain	Genotype	Source
PDS0066	*B. subtilis* NCIB 3610 wild type	Laboratory collection
PDS0934	*B. subtilis* NCIB 3610 Δ*bmrCD:kan*	This study
PDS0986	*B. subtilis* NCIB 3610 Δ*vmlR*	This study
PDS0987	*B. subtilis* NCIB 3610 Δ*yxjB*	This study
PDS0988	*B. subtilis* NCIB 3610 Δ*mdr*	This study
PDS0989	*B. subtilis* NCIB 3610 Δ*ytbD*	This study
PDS0991	*B. subtilis* NCIB 3610 Δ*5:: kan(Δ vmlR, ΔbmrCD::kan, ΔyxjB, Δmdr, ΔytbD)*	This study
PDS0992	*B. subtilis* NCIB 3610 Δ*amyE*::P*hy-bmrCD-spec*	This study
PDS0993	*B. subtilis NCIB 3610 ΔbmrCD::kan ΔamyE::Phy-bmrCD-spec*	This study
PDS0994	*B. subtilis NCIB 3610 ΔamyE::Phy-bmrC*D*-spec (For bmrC, mutate AAA to GCA; K377A; For bmrD, mutate AAA to GCT; K469A)*	This study
PDS0995	*B. subtilis NCIB 3610 ΔbmrCD::kan ΔamyE::Phy-bmrC*D*-spec (For bmrC, mutate AAA to GCA; K377A For bmrD, mutate AAA to GCT;K469A)*	This study
PDS1216	*B. subtilis NCIB 3610 ΔamyE::PbmrCD-luxABCDE-kan*	This study
PDS1268	*B. subtilis NCIB 3610 ΔamyE::PvmlR-luxABCDE-kan*	This study
PDS0969	*B. subtilis NCIB 3610 ΔamyE::PtlrB-luxABCDE-kan*	This study

Table S3. Primers used in this study.

Primer	Sequence (5'-3')
lux-For	AGGAGGCTAGCCTATGAAATTTGGAAACTTTTTGC
lux-Rev (overhang paired with kan cassette)	CACCTCAAATGGTTCGCTGTCAACTATCAAACGCTTC
amyE-back-For	ATCCGTTTAGGCTGGGC
amyE-front-Rev	TGTACTATTTCGATCAGACC
PbmrCD-For	GTCTGATCGAAATAGTACAATACGGTCAGCATGGAACAT
P*bmrCD*-Rev (overhang)	ATTTCATAGGCTAGCCTCCCTATCCCCATCTCCTCAT
kan-For-new	CAGCGAACCATTTGAGGTGA
kan-Rev-new	CGATACAAATTCCTCGTAGGCG
P*yxjB*-For	GGTCTGATCGAAATAGTACAACTGCTGACGTTAATGCG
P*yxjB*-Rev	ATTTCATAGGCTAGCCTCCTGATAAAAAACACCCCATTCAT
P*vmlR*-For	GGTCTGATCGAAATAGTACAGACTCAAAACTCCTGCCTC
P*vmlR*-Rev	ATTTCATAGGCTAGCCTCCTCCATATCCCTCGCTTTAAAG
spec-fwd	CAACGTTCTTGCCATTGC
spec-rev	GATCCCCCTATGCAAGGGT
RBS+*bmrC*-fwd	aggaggctagcctATGTTTTCAGTTTTGAAAAAGC
bmrD-rev	CAGCAATGGCAAGAACGTTGTTATGCAATGGAATGTTTCTG
bmrC1-mutation-rev	TTGTCGTTGCTCCGCTCCCGGTTTTAC
bmrC2-mutation-fwd	CGGGAGCGGAGCAACGACAATTATTAAGCAGC
bmrD1-mutation-rev	TCGAGCTAGCTCCTGATCCGGTATGGC
bmrD2-mutation-fwd	CGGATCAGGAGCTAGCTCGATTTTGAATCTTC
lacI-fwd	GGTCTGATCGAAATAGTACACATGCAAGCTAATTCGGTGG
lacI-rev	TGCTCACATTTaccctcgagTAATGGATTTCCTTACGCGA
P*hy*-fwd	ctcgagggtAAaTGTGAGCa
P*hy*-rev	AAAACATAGGCTAGCCTCCTAAGCTTAATTGTTATCCGCTC

amyE-Back-fwd	AACCCTTGCATAGGGGGATCATCCGTTTAGGCTGGGCTGTACTATTTCGATCAGACC
amyE-Front-rev	TGTACTATTTCGATCAGACC
